# Highly Efficient Adsorption of Uranium(VI) Ions in Aqueous Solution by Imidazole-4,5-Dicarboxylic Acid-Functionalized UiO-66

**DOI:** 10.3390/molecules30142966

**Published:** 2025-07-15

**Authors:** Tian Lan, Xiechun Liu, Haifeng Cong, Xiaofan Ding, Jing Zhao, Songtao Xiao

**Affiliations:** Department of Radiochemistry, China Institute of Atomic Energy, Beijing 102413, China; lant3@163.com (T.L.); henryliu99@163.com (X.L.); 79819cong@163.com (H.C.); zhuozhu15@126.com (J.Z.)

**Keywords:** metal-organic framework, imidazole-4,5-dicarboxylic Acid, UiO-66(Zr), high uranium adsorption capacity, high selectivity

## Abstract

In this study, a novel adsorbent, UiO-66-H_3_IMDC, was successfully prepared by functionalizing UiO-66 with imidazole-4,5-dicarboxylic acid (H3IMDC). The effective functionalization of H_3_IMDC on UiO-66 was confirmed by powder X-ray diffraction (PXRD) and Fourier transform infrared spectroscopy (FT-IR). The relationships between the adsorption of U(VI) on UiO-66-H_3_IMDC and the contact time, the pH of the solution, as well as the initial concentration of U(VI) were investigated. Additionally, the selective adsorption of U(VI) by UiO-66-H_3_IMDC and its cyclic regeneration performance were also studied. The results demonstrate that the UiO-66-H_3_IMDC adsorbent exhibits excellent adsorption performance for uranium in aqueous solutions.

## 1. Introduction

During uranium mining, nuclear fuel processing, nuclear power generation, and spent fuel reprocessing, substantial amounts of uranium-containing radioactive waste are generated [1]. Upon entering the human body, uranium primarily accumulates in the liver, kidneys, and skeletal system, potentially causing acute/chronic poisoning, multi-organ diseases, hepatic damage, and carcinogenic effects. Effective management of such radioactive waste is imperative, with uranium-contaminated wastewater treatment representing a critical component of radwaste disposal [2]. Currently, the treatment methods are divided into three major categories: chemical methods [3], physical-chemical methods [4], and biological methods [5]. Specific treatment technologies include traditional methods such as evaporation concentration [6], chemical precipitation [7], ion exchange [8], electrochemical methods [9], and microbial treatment [10]. With the development of materials science, new types of adsorbent materials keep emerging, and the treatment of radioactive wastewater by the adsorption method has received special attention in recent years [11].

Metal-organic frameworks (MOFs) are a class of porous crystalline materials composed of organic ligands connected via coordination bonds [12]. These extensively studied materials demonstrate exceptional potential for radioactive wastewater remediation due to their unique combination of properties, including tunable pore architectures, structural customizability, and high crystallinity. While pristine MOFs typically exhibit limited intrinsic adsorption capacities for radionuclides, targeted modifications have been shown to significantly enhance their adsorption performance. This optimization enables more efficient removal of radioactive species such as uranyl ions (UO_2_^2+^) from contaminated water systems [13,14,15,16,17].

Understandably, MOFs serving as adsorbents require stability in aqueous or acidic environments. A breakthrough came with Lillerud and colleagues’ synthesis of a zirconium(IV) dicarboxylate porous material, designated as UiO-66 [18]. This framework demonstrated exceptional surface area and unprecedented chemical stability, establishing a new benchmark in the field. Subsequent research efforts have extensively investigated the uranyl ion (UO_2_^2+^) adsorption capabilities of both pristine UiO-66 and its structural analogs, driving innovations in nuclear waste treatment technologies. Luo et al. [19] studied the ability of UIO-66-NH_2_ to capture U(VI) from an aqueous solution. Under the condition of pH 5.5, the adsorption of U(VI) reached equilibrium in approximately 4 h, and the maximum adsorption capacity was 114.9 mg g^−1^. RAJAEI et al. [20] studied the modification of UIO-66 and vacant UIO-66 (UiO-66-vac) by immobilizing tributyl phosphate (TBP), and these modified materials were used for the removal of uranyl ions from an aqueous solution. The research results showed that the maximum adsorption capacities of UiO-66-TBP and UiO-66-vac-TBP for uranium were 201.9 and 203.5 mg g^−1^, respectively. That is, the immobilization of TBP significantly enhanced the adsorption capacity of MOFs for uranyl ions. It can be seen that although researchers have carried out various modifications on UiO-66 to improve its uranium adsorption capacity, the results are not entirely satisfactory. Therefore, on the basis of taking into account the high chemical stability of UiO-66, it is of great significance to explore how to develop a functionalized UiO-66 adsorbent with a high adsorption capacity.

Imidazole-4,5-dicarboxylic acid (H_3_IMDC) is a versatile ligand containing imidazole nitrogen and two carboxyl groups [21]. Its molecular structure features two carboxylic acid groups and an imidazole ring nitrogen atom, which together provide multiple coordination sites to form stable chelates with metal ions [22]. Metal complexes derived from this ligand exhibit strong stability under high-temperature and chemically harsh environments, making them advantageous for the regeneration and reuse of adsorbent materials [23]. Furthermore, the amphoteric nature of the imidazole ring—capable of protonation/deprotonation—enables it to maintain adsorption activity across a broad pH range, allowing adaptability to diverse wastewater treatment conditions [24]. This unique combination of structural and chemical properties positions imidazole-4,5-dicarboxylic acid as a promising candidate for designing robust and reusable materials for environmental remediation applications [25,26,27].

Therefore, in this study, UiO-66(Zr) was functionalized with H_3_IMDC to enhance its uranium adsorption performance. Compared to pristine UiO-66 and H_3_IMDC individually, the UiO-66-H_3_IMDC composite exhibited a significant improvement in U(VI) ion adsorption capacity. Key factors influencing adsorption behavior—including pH, initial U(VI) concentration, contact time, ionic competition, and regeneration cycles—were systematically investigated. Furthermore, Fourier transform infrared (FT-IR) spectroscopy was employed to elucidate the adsorption mechanism of UiO-66-H_3_IMDC.

## 2. Results and Discussion

### 2.1. Characterization of MOFs

**Figure 1:**
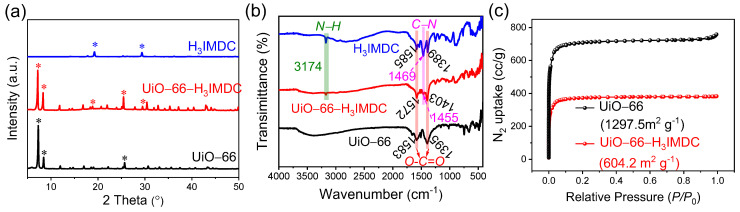
(**a**) PXRD pattern and (**b**) infrared spectra of UiO-66, UiO-66-H_3_IMDC, and pristine H_3_IMDC and (**c**) N_2_ adsorption-desorption isotherm at 77 K of UiO-66 and UiO-66-H_3_IMDC. (* * *: Labeling of characteristic peak positions in PXRD).

Figure 1a displays the PXRD patterns of UiO-66, UiO-66-H_3_IMDC, and pristine H_3_IMDC. The characteristic diffraction peaks of both UiO-66 and H_3_IMDC are distinctly present in the UiO-66-H_3_IMDC composite. This dual observation not only confirms the successful synthesis of crystalline UiO-66-H_3_IMDC but also demonstrates the effective functionalization of UiO-66 through H_3_IMDC incorporation. Additionally, Figure 1b presents the Fourier transform infrared (FT-IR) spectra of pristine UiO-66, H_3_IMDC, and UiO-66-H_3_IMDC. The absorption peak at 3174 cm^−1^ corresponds to N-H stretching vibrations inherent in the pure H_3_IMDC ligand [25]. Following H_3_IMDC modification, significant spectral shifts emerge in two critical regions: both the C-N (1469–1457 cm^−1^) and O-C=O (1585–1578 cm^−1^, 1389–1397 cm^−1^) characteristic peaks exhibit notable displacement compared to their original positions in pure H_3_IMDC. And the O-C=O vibrational signature in UiO-66-H_3_IMDC (1397 cm^−1^, 1578 cm^−1^) displays marked deviation from its counterpart in pristine UiO-66 (1395 cm^−1^, 1583 cm^−1^) [28]. These spectral discrepancies collectively confirm the successful covalent grafting of H_3_IMDC onto the UiO-66 framework. The N_2_ adsorption-desorption isotherms of UiO-66 and UiO-66-H_3_IMDC at 77 K exhibit classical Type I behavior (Figure 1c), confirming their microporous architectures. Post functionalization, UiO-66-H_3_IMDC demonstrates a substantial reduction in BET surface area (from 1297.5 m^2^ g^−1^ to 604.2 m^2^ g^−1^), primarily due to partial pore blockage caused by H_3_IMDC grafting. This steric hindrance effect arises from the chelation of H_3_IMDC carboxylate groups to Zr_6_O_4_(OH)_4_ clusters, which modifies the pore accessibility while preserving the overall framework crystallinity.

**Figure 2:**
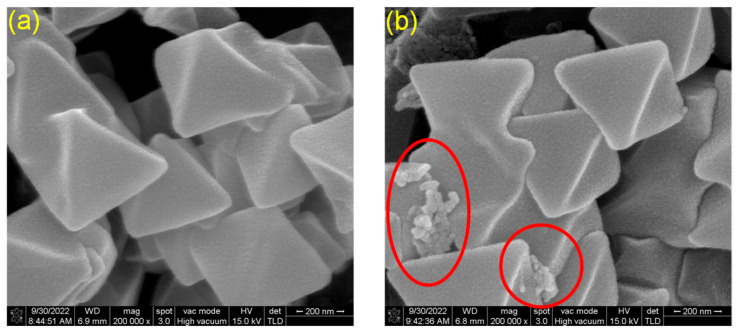
SEM images of (**a**) UiO-66 and (**b**) UiO-66-H_3_IMDC.

Furthermore, Figure 2 displays the morphological characteristics of UiO-66 and UiO-66-H_3_IMDC, respectively. As can be observed, UiO-66 exhibits a smooth surface morphology. Upon grafting H_3_IMDC onto UiO-66, the crystal structure of the parent material remains intact and undamaged. Notably, irregular particles become distinctly visible and dispersed across the surface of UiO-66-H_3_IMDC, as highlighted by the circular markers. Further SEM mapping investigated the distribution of carbon (C), oxygen (O), nitrogen (N), and zirconium (Zr) within the sample (Figure S1a–e). Analysis revealed that these elements constituted approximately 58.19%, 10.50%, 29.94%, and 1.38% of the sample composition, respectively (Table S1).

### 2.2. Effect of Initial pH

**Figure 3:**
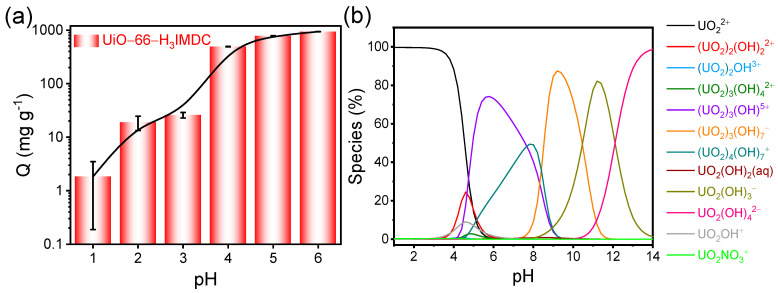
(**a**) Effect of pH on UiO-66-H_3_IMDC on U (VI) adsorption (adsorbent dosage = 3 mg; C_0_ = 300 mg L^−1^; *t* = 8 h; T = 298.15 K). (**b**) Variation in Th (IV) species with pH of aqueous solution (Visual Minteq-3.1 program).

Given that solution pH significantly influences the protonation and deprotonation of functional groups on the adsorbent surface, as well as the speciation of metal ions in solution [29], we prioritized exploring the impact of pH on our adsorption experiments. Figure 3a illustrates the adsorption outcomes of UiO-66-H_3_IMDC for U(VI) at various pH levels. Notably, the adsorption capacity for U(VI) is relatively low at low pH values. This can be attributed to the high concentration of H^+^ ions in the solution at low pH, which compete with U(VI) ions for adsorption sites on the UiO-66-H3IMDC surface. On the other hand, pH-induced U(VI) speciation may also account for the pH-dependent adsorption. It is well-known that as the pH increases, the U(VI) species gradually transform from free UO_2_^2+^ to polynuclear hydroxide complexes (Figure 3b). These hydroxide complexes are likely to be more favorably adsorbed by the adsorbent. In the subsequent experiments, a pH of 6.0 was selected as an appropriate condition for further investigation.

### 2.3. Adsorption Isotherm

**Figure 4:**
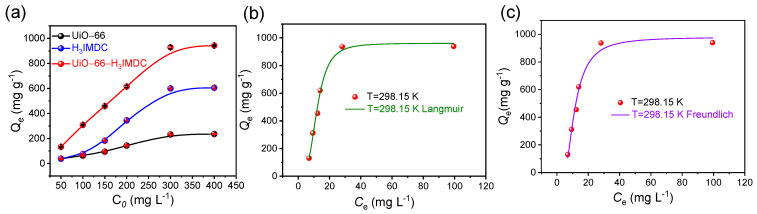
(**a**) Adsorption isotherms of UiO-66, H_3_IMDC, and UiO-66-H_3_IMDC (T = 298.15 K, *t* = 8 h; pH = 6.0). (**b**) Langmuir model and (**c**) Freundlich model of UiO-66-H_3_IMDC.

The uranium sorption performance of UiO-66, H_3_IMDC, and UiO-66-H_3_IMDC was systematically evaluated. As shown in Figure 4a, the adsorption isotherms of these three materials exhibit a significant enhancement in uranium uptake capacity after functionalization. The maximum sorption capacities of UiO-66, H_3_IMDC, and UiO-66-H_3_IMDC within the tested concentration range were determined as 235.5, 605.3, and 942.8 mg g^−1^, respectively. UiO-66-H_3_IMDC demonstrates exceptional uranyl ion (UO_2_^2+^) adsorption capacity that significantly surpasses most MOF-based adsorbents reported in the current literature (Table S2). Despite the substantially reduced BET surface area of the functionalized UiO-66 (attributed to pore-blocking effects from H_3_IMDC coordination), the integration of H_3_IMDC introduced high-efficiency binding sites (e.g., carboxylate and imidazole groups), enabling exceptional uranium affinity that compensates for porosity loss.

To elucidate the sorption mechanism, the adsorption data were fitted with two classical isotherm models, the Langmuir and Freundlich models [30,31].

Langmuir isotherm equation:

(1) $$\frac{C_{e}}{Q_{e}}=\frac{1}{K_{L}Q_{m}}+\frac{C_{e}}{Q_{m}}$$

Freundlich isotherm equation:

(2) $$lnQ_{e}=ln+lnC_{e}$$

where *C_e_* (mg L^−1^) denotes the equilibrium U(VI) concentration in the aqueous phase, *Q_e_* (mg g^−1^) represents the equilibrium adsorption capacity, and *Q_m_* (mg g^−1^) corresponds to the theoretical maximum adsorption capacity derived from the Langmuir isotherm model. The parameter *K*_L_ is the Langmuir affinity constant, while *K*_F_ and n are the Freundlich constants characterizing adsorption capacity and heterogeneity, respectively.

The experimental versus modeled adsorption profiles for the Langmuir and Freundlich isotherms are comparatively visualized in Figure 4b,c, with detailed fitting parameters and correlation coefficients (R^2^) tabulated in Table S3. Notably, the Langmuir model demonstrates superior agreement (R^2^ = 0.992) over the Freundlich model (R^2^ = 0.984). This pronounced preference for the Langmuir isotherm strongly supports a monolayer adsorption mechanism governed by homogeneous active sites across the adsorbent surface [32,33,34,35,36,37], where U(VI) species undergo chemo selective coordination with the imidazole-dicarboxylate functionalities of UiO-66-H_3_IMDC.

### 2.4. Adsorption Kinetics

**Figure 5:**
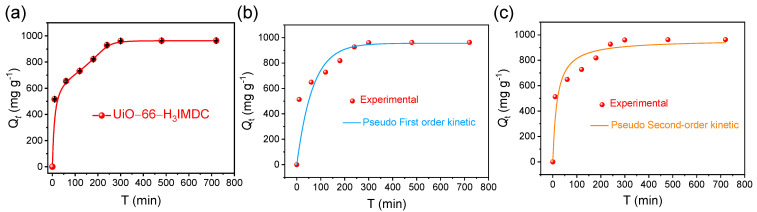
(**a**) Effect of uranium adsorption time on UiO-66-H_3_IMDC (C_0_ = 300 mg L^−1^; T = 298.15 K; pH = 6.0); (**b**) pseudo-first-order model and (**c**) pseudo-second-order model fitting curves.

The adsorption kinetics were investigated through time-dependent batch experiments, with the temporal evolution of adsorption capacity quantified in Figure 5a. Kinetic analysis revealed a triphasic adsorption mechanism: (1) an initial rapid adsorption stage (0–100 min) dominated by surface complexation, (2) a transitional diffusion-controlled phase (100–300 min) showing progressive site saturation, and (3) an equilibrium plateau (>300 min) achieving maximum adsorption capacity. Notably, >60% of total uranium uptake occurred within the first kinetic phase, followed by gradual pore-filling processes until complete monolayer formation.

The interfacial mass transfer mechanisms governing U(VI) adsorption on UiO-66-H_3_IMDC were elucidated through kinetic modeling using two classical formulations [38,39]: the pseudo-first-order-model (PFO) Equation (3) for physisorption-dominated processes and the pseudo-second-order model (PSO) Equation (4) describing chemisorption-controlled systems. The linearized rate equations are mathematically expressed as

ln(*Q_e_* − *Q_t_*) = ln*Q_e_* − *k*_1_*t* (3)

(4) $$\frac{t}{Q_{t}}=\frac{1}{k_{2}Q_{e}^{2}}+\frac{t}{Q_{e}}$$

where *k*_1_ (min^−1^) and *k*_2_ (g mg^−1^ min) are the adsorption constants. *Q_e_* and *Q_t_* denote the amount of adsorption (mg g^−1^) at the equilibrium moment and t (min) moment, respectively.

Figure 5b,c present the nonlinear regression analysis of kinetic models, with corresponding goodness-of-fit statistics detailed in Table S4. The pseudo-second-order (PSO) model demonstrates superior predictive capability, evidenced by its coefficient of determination (R^2^ = 0.927) significantly exceeding that of the pseudo-first-order (PFO) model (R^2^ = 0.796). This high degree of correlation establishes through rigorous statistical validation that the U(VI) adsorption process on UiO-66-H_3_IMDC follows PSO kinetics, indicative of rate-limiting chemisorption mechanisms potentially involving surface complexation or ion-exchange reactions [40,41,42].

### 2.5. Effect of Co-Existing Ions

**Figure 6:**
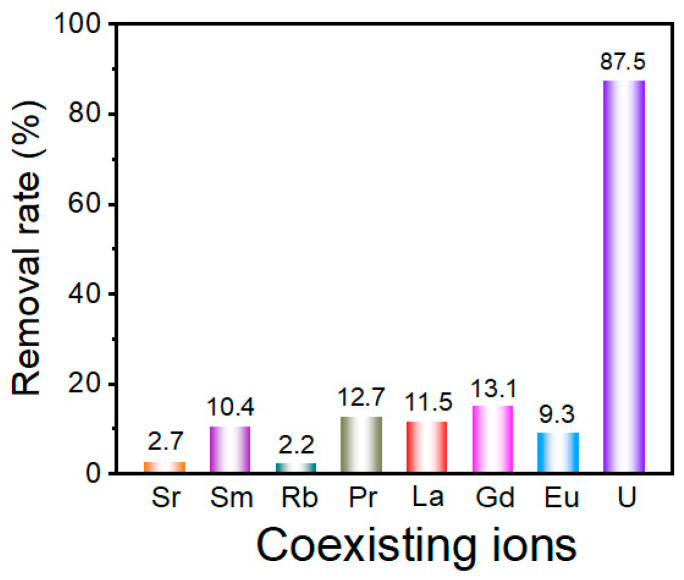
The effect of interfering ions on adsorption capacity for Th (IV) ion on UiO-66-H_3_IMDC. (C^M+^ = 300 mg L^−1^; *t* = 8 h; pH = 6.0; T = 298.15K).

The remediation of U(VI) from radioactive wastewater necessitates adsorbents with exceptional ion-discrimination capability, given the inherent challenges posed by heterogeneous ionic matrices containing competing cations. In this study, we ascertained the selective adsorption capabilities of U(VI) in multi-ionic solutions, and the obtained results are presented in Figure 6. After the adsorption process, the concentration of U(VI) ions in the coexisting solution is markedly lower than that of other coexisting ions. Notably, the presence of interfering ions does not impede the adsorption of U(VI) by UiO-66-H_3_IMDC. Thus, this material may be used in the treatment of real radioactive wastewater.

### 2.6. Regeneration and Stability Investigation

**Figure 7:**
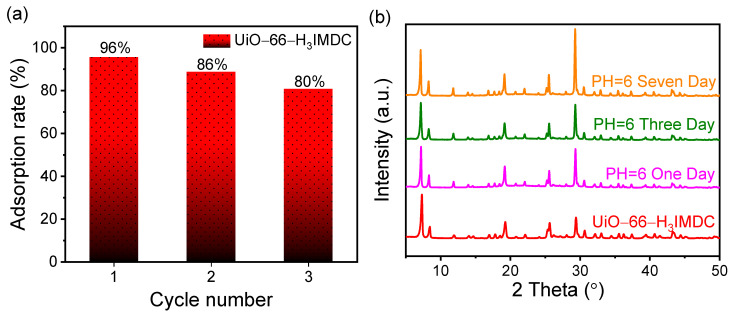
(**a**) Cyclic regeneration and (**b**) water stability of UiO-66-H_3_IMDC.

In practical scenarios, guaranteeing the reusability and stability of adsorbents is essential. As a result, both the adsorption efficiency and the adsorption capacity throughout the adsorption/desorption cycle have emerged as crucial benchmarks for assessing the performance of adsorbents. As illustrated in Figure 7a, following three regeneration cycles, the adsorption quantity witnesses a 16% reduction. Significantly, the adsorption rates continue to stay elevated throughout this process. Significantly, UiO-66-H_3_IMDC maintained structural integrity with indistinguishable PXRD patterns from the pristine material after 7-day immersion in aqueous media (pH = 6) (Figure 7b), providing robust evidence of its exceptional hydrolytic stability under environmentally relevant conditions.

### 2.7. Removal Mechanism

**Figure 8:**
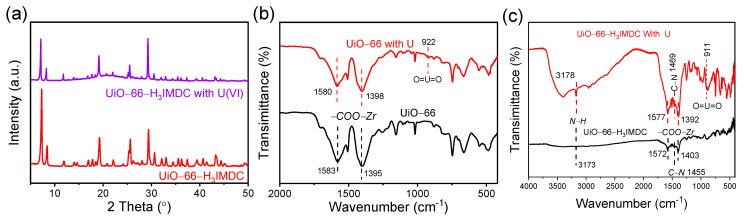
(**a**) PXRD of UiO-66-H_3_IMDC before and after uranium adsorption. FT-IR spectra of (**b**) UiO-66 and (**c**) UiO-66-H_3_IMDC before and after uranium adsorption.

Following the adsorption of U(VI), the PXRD patterns of the UiO-66-H_3_IMDC show diffraction peaks that precisely match those of the original materials (Figure 8a). This result clearly demonstrates that the crystal structure remains intact during the uranium adsorption process. To further investigate the mechanism of strong chemical interaction between U(VI) ions and UiO-66-H_3_IMDC, we compared the infrared (IR) spectral shifts of UiO-66 and UiO-66-H_3_IMDC before and after uranium adsorption (Figure 8b,c). Post adsorption, new O=U=O vibrational bands appeared at 922 cm^−1^ and 911 cm^−1^ [43], directly confirming successful uranium coordination on the material’s surface. Notably, the O=U=O peak associated with H_3_IMDC exhibited a lower vibrational frequency compared to UiO-66, suggesting a stronger adsorption of U(VI) by H_3_IMDC. Additionally, the -COO-Zr doublet peaks in UiO-66-H_3_IMDC (1582–1577 cm^−1^, 1405–1392 cm^−1^) underwent significantly larger shifts than those in UiO-66 (1583–1580 cm^−1^, 1395–1398 cm^−1^), further evidencing enhanced chemical interactions between UiO-66-H_3_IMDC and U(VI), consistent with its superior adsorption capacity. Furthermore, significant shifts in the N-H (3173–3718 cm^−1^) and C-N (1457–1469 cm^−1^) regions indicate that the nitrogen adsorption sites on UiO-66-H_3_IMDC also interact with U(VI) ions. The mechanism may be as follows: Firstly, the large specific surface area and abundant porous structure of the UiO-66-H_3_IMDC material enable U(VI) species to efficiently diffuse and come into full contact with the composite material. Subsequently, uranyl ions coordinate with the nitrogen- and oxygen-containing active adsorption sites. Therefore, uranium(VI) can not only interact with the surface of the adsorbent but also undergo complexation with more reactive sites. Additionally, we selected the two ions exhibiting the highest (gadolinium) and lowest (rubidium) removal rates among the competing ions besides uranium to explore whether strong chemical interactions exist between these competing ions and UiO-66-H_3_IMDC (Figure S2). Results revealed no shifts in the characteristic FTIR peaks corresponding to the adsorption sites (N-H, COO-Zr, C-N) for both rubidium (Rb^+^) and gadolinium (Gd^3+^) ions before and after adsorption. This observation further confirms the high adsorption selectivity of UiO-66-H_3_IMDC for uranium.

## 3. Materials and Methods

### 3.1. Materials

Zirconyl chloride octahydrate (ZrOCl_2_·8H_2_O, AR), terephthalic acid (H_2_BDC, >99.0%), 4,5-Imidazoledicarboxylic acid (C_5_H_4_N_2_O_4_, AR), Acetate (C_2_H_4_O_2_, AR), N, N-Dimethylformamide (DMF, >99.5%), and methanol (CH_3_OH, AR) were purchased from MACKLIN reagent (Shanghai, China). Strontium nitrate (SrN_2_O_6_, AR), cesium nitrate (CsNO_3_, AR), samarium nitrate hexahydrate (SmN_3_O_9_·6H_2_O, AR), rubidium nitrate (RbNO_3,_ AR), praseodymium nitrate hexahydrate (PrN_3_O_9_·6H_2_O, AR), lanthanum nitrate hexahydrate (LaN_3_O_9_·6H_2_O, AR), gadolinium nitrate hexahydrate (GdN_3_O_9_·6H_2_O, AR), and europium nitrate hexahydrate (EuN_3_O_9_·6H_2_O, AR) were purchased from Aladdin (Shanghai, China). Uranyl nitrate hexahydrate (UO_2_(NO_3_)_2_·6H_2_O, AR) was obtained from the China Institute of Atomic Energy. Ultrapure water was prepared from the Millipore system (18.25 MΩ cm) (Direct 8, Millipore, Burlington, MA, USA). All the above reagents were used directly without further purification.

### 3.2. Preparation of UiO-66(Zr)

UiO-66(Zr) was synthesized by a solvothermal synthesis technique reported in the literature [18]. H_2_BDC (1.6 g) and ZrOCl_2_·8H_2_O (3.2 g) were dissolved in DMF (50 mL)/acetate (50 mL) and mixed completely, then transferred to a 250 mL round-bottomed flask and stirred at 378.15 K for 24 h under reflux in an oil bath. After that, white powder was obtained through centrifugation. Finally, the sample was washed three times with DMF and three times with methanol and then dried in a vacuum drying oven to obtain the sample.

### 3.3. Preparation of UiO-66-H_3_IMDC

UiO-66 (Zr) (0.5 g) and H_3_IMDC (1.0 g) were dissolved in a solvent mixture of 100 mL ethanol and 50 mL DMF. The mixture was sonicated for 20 min to ensure uniform dispersion. After sonication, the solution was subjected to reflux heating at 353.15 K for 24 h. Upon cooling to room temperature, the product was separated via centrifugation, followed by washing the solid product three times with deionized water and ethanol. Finally, the material was vacuum-dried overnight to yield the functionalized UiO-66-H_3_IMDC composite.

### 3.4. Characterization Techniques

Powder X-ray diffraction (PXRD) analysis was conducted on a Bruker D8 QUEST diffractometer (Bremen, Germany) to evaluate the crystallinity and phase purity of the MOF materials. Measurements utilized Cu Kα radiation (*λ* = 1.542 Å) operated at 40 kV and 40 mA, with a scan range of 4–50.0° (2*θ*) at a rate of 10° min^−1^. For porosity characterization, nitrogen adsorption-desorption isotherms were recorded at 77 K using a Micromeritics ASAP 2460 analyzer (Norcross, GA, USA). Prior to analysis, MOF samples (~100 mg) were degassed under vacuum at 80 °C for 12 h to remove physisorbed species. Specific surface areas were calculated via the Brunauer-Emmett-Teller (BET) method. Chemical bonding analysis was performed by Fourier transform infrared spectroscopy (FT-IR) on a Bruker TENSOR27 spectrometer (Germany). Spectra were acquired in the 400–4000 cm^−1^ range to monitor functional group transformations in MOFs. The morphology of the materials was observed using a scanning electron microscope (SEM) (JEM 2100, JEOL, Akishima, Japan) operating at 30.0 kV under high vacuum conditions. The sample was vacuum-dried and affixed to the test bench with a conductive adhesive before gold sputtering for 80 s to facilitate microscopic observation.

### 3.5. Adsorption Experiments

Batch adsorption experiments were performed by adding 3 mg of adsorbent to 10 mL of U(VI) solution in 15 mL polypropylene centrifuge tubes. The suspensions were agitated at 220 rpm in a temperature-controlled orbital shaker for predetermined time intervals. Subsequently, the mixtures were filtered through 0.22 μm pore-size nylon membranes using a syringe filtration assembly (5 mL capacity). The collected filtrates were acidified with 2% (*v*/*v*) HNO_3_ and analyzed for residual U(VI) concentrations via inductively coupled plasma optical emission spectrometry (ICP-OES, JY2000-2, HORIBA, Bhamboli, Maharashtra). Specific experimental parameters (pH, initial U(VI) concentration, and contact time, etc.) are provided in the captions of relevant figures.

The adsorption capacity (*Q*) of U(VI) was defined by the following equation:

(5) $$Q=\frac{\left(C_{0}-C_{e}\right)\times V}{m}$$

where *C*_0_ (mg L^−1^) and *C_e_* (mg L^−1^) are the initial concentration and equilibrium concentration of U(VI) ions, respectively. *V* (L) is the volume of solution, and *m* (g) is the usage amount of adsorbent.

## 4. Conclusions

In conclusion, a novel MOF (UiO-66-H_3_IMDC) was successfully synthesized through grafting H_3_IMDC onto UiO-66, demonstrating exceptional U(VI) adsorption capabilities. The modified material exhibited a maximum U(VI) adsorption capacity of 942.8 mg g^−1^ under optimized conditions (pH = 6), reaching adsorption equilibrium within 300 min. Additionally, the uranium adsorption process of UiO-66-H_3_IMDC fits better with the Langmuir model and the pseudo-second-order model, indicating that the adsorption process is predominantly a monolayer chemisorption process. Finally, the excellent selectivity and cyclic adsorption ability demonstrated by UiO-66-H_3_IMDC suggest that UiO-66-H_3_IMDC has the potential to adsorb U(VI) from actual wastewater.

## Data Availability

Dataset available on request from the authors.

## Supplementary Materials

The following supporting information can be downloaded at: https://www.mdpi.com/article/10.3390/molecules30142966/s1, Figure S1: (a) SEM image of UiO-66-H_3_IMDC; (b–e) SEM-EDS mapping of elements C, O, N, and Zr in UiO-66-H_3_IMDC. Figure S2: FT-IR spectra of UiO-66-H_3_IMDC before and after rubidium and gadolinium adsorption. Table S1: Elemental distribution of UiO-66-H_3_IMDC. Table S2: Uranium adsorption capacity of MOFs and their materials. Table S3: Langmuir and Freundlich model fitting parameters for U (VI) adsorption. Table S4: Kinetic fitting parameters of U (VI) adsorption in UiO-66-H_3_IMDC. Refs. [13,14,15,16,17,19,20,43,44,45,46,47,48,49] are cited in Supplementary Materials.
