# Supplementary material for: Highly Efficient Adsorption of Uranium(VI) Ions in Aqueous Solution by Imidazole-4,5-Dicarboxylic Acid-Functionalized UiO-66
Source: Molecules. 2025 Jul 15;30(14):2966. doi: 10.3390/molecules30142966 (PMC12298460; doi:10.3390/molecules30142966)
Supplement: Supplementary file 1 [file molecules-30-02966-s001.zip › molecules-3690173-supplementary.pdf]

Supplementary information

Highly Efficient Adsorption of Uranium(VI) Ions in Aqueous Solution by Imidazole – 4,5 – dicarboxylic Acid Functionalized UiO–66

Tian Lan, Xiechun Liu, Haifeng Cong, Xiaofan Ding*, Jing Zhao, Songtao Xiao*

Department of Radiochemistry, China Institute of Atomic Energy, Beijing 102413, China

*Corresponding author. email: xiaofan_ding@163.com (X.D.); xiao_songtao@126.com (S. X.);

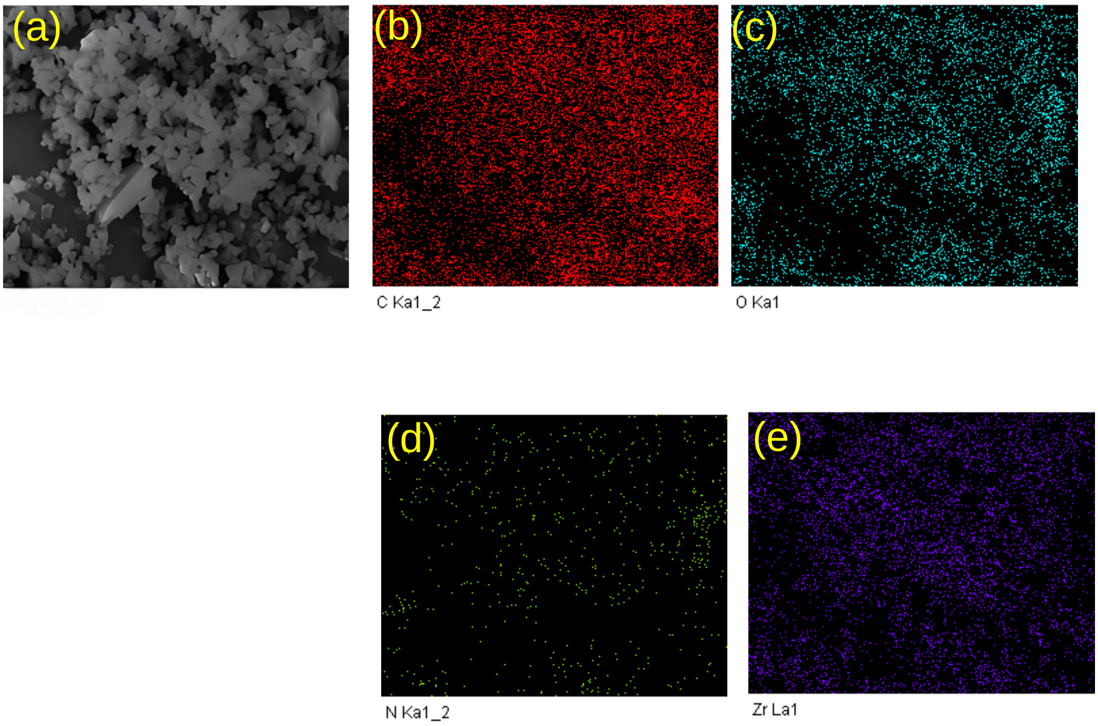

Figure S1 (a) SEM image of UiO–66–H_3IMDC; (b–e) SEM–EDS–mapping of elements C, O, N and Zr in UiO–66–H_3IMDC

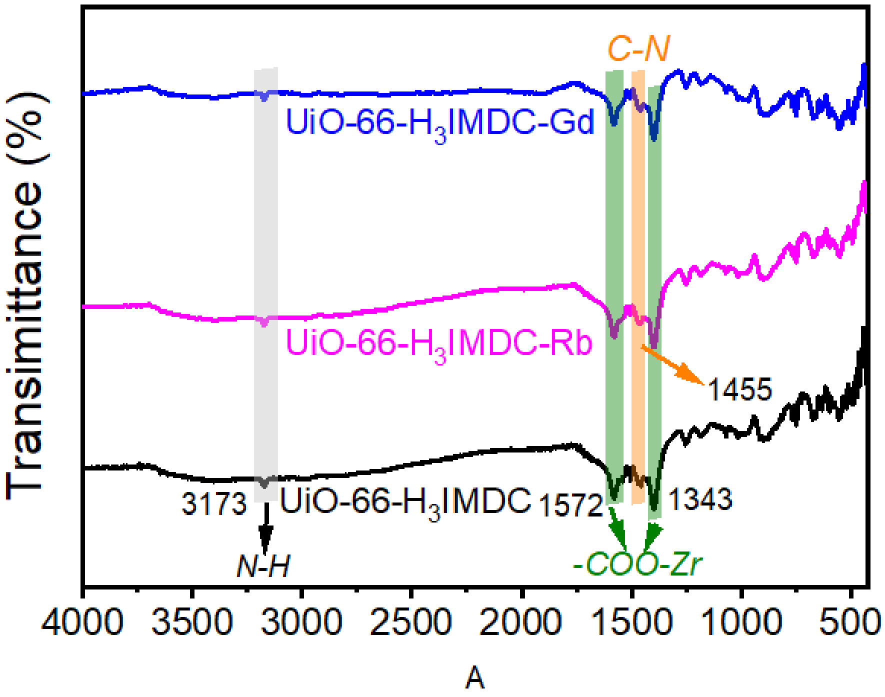

Figure S2 FT–IR spectra of UiO–66–H3IMDC before and after rubidium and gadolinium adsorption.

Table S1 Elemental distribution of UiO–66–H_3IMDC

Element	At%
C	58.19
N	10.50
O	29.94
Zr	1.38

Table S2 Uranium adsorption capacity of MOFs and their materials

MOFs	pH	Capacity (mg g–1)	Ref.
MIL–101–ship	4	27.99	1
MIL–101–SMA–AO	7	847	2
DSHM–DAMN	8	601	3
MIL–101–AO	7	550	4
MIL–100(Fe)	10	210~270	5
UiO–66–NH_2	5.5	114.9	6
UiO–66–vac–TBP	5	203.5	7
UiO–66@PAO	6	579	8
UiO–66–2COOH	4	150	9
GO–COOH/UiO–66	8	188.3	10
ZIF–67	4	397.6	11
PCN–222–AO	7	432.7	12
P(AM–co–AA)/CS/MOF–808	8	159.56	13
DNPM–3	6	334.67	14
UiO–66–H_3IMDC	6	942.8	This work

Table S3 Langmuir and Freundlich model fitting parameters for U (VI) adsorption

Model	Parameters	T (K)
		298
Langmuir	Q_{max} (mg/g)	961.8
	K_L (L/mg)	0.1268
	R^2	0.992
Freundlich	K_F(mg^{1-n}•L^n/g)	300.53
	n	0.7951
	R^2	0.984

Table S4 Kinetic fitting parameters of U (VI) adsorption in UiO–66–H_3IMDC

Kinetic model	T	C_0	k_1/k_2	R^2
	K	(mg L^{-1})	(min^{-1})/(g/mg·min)	
Pseudo–first order	298	300	0.025771	0.796
Pseudo–second order	298	300	0.000167	0.927

References

[1] J. De Decker, K. Folens, J. De Clercq, M. Meledina, G. Van Tendeloo, G. Du Laing, P. Van Der Voort, Ship–in–a–bottle CMPO in MIL–101(Cr) for selective uranium recovery from aqueous streams through adsorption, J. Hazard. Mater., **2017**, 335, 1–9.

[2] Y. Meng, Y. Wang, L. Liu, F. Ma, C. Zhang, H. Dong, MOF modified with copolymers containing carboxyl and amidoxime groups and high efficiency U (VI) extraction from seawater, Sep. Purif. Technol., **2022**, 291, 120946.

[3] J. Zhang, H. Zhang, Q. Liu, D. Song, R. Li, P. Liu, J. Wang, Diaminomaleonitrile functionalized double–shelled hollow MIL–101 (Cr) for selective removal of uranium from simulated seawater, Chem. Eng. J., **2019**, 368, 951–958.

[4] L. Liu, Y. Fang, Y. Meng, X. Wang, F. Ma, C. Zhang, H. Dong, Efficient adsorbent for recovering uranium from seawater prepared by grafting amidoxime groups on chloromethylated MIL–101(Cr) via diaminomaleonitrile intermediate, Desalination, **2020**, 478, 114300.

[5] A. Das, D. Roy, K. Erukula, S. De, Synthesis of pH responsive malononitrile functionalized metal organic framework MIL–100(Fe) for efficient adsorption of uranium U(VI) from real–life alkaline leach liquor, Chemosphere, **2024**, 348, 140780.

[6] B.–C. Luo, L.–Y. Yuan, Z.–F. Chai, W.–Q. Shi, Q. Tang, U(VI) capture from aqueous solution by highly porous and stable MOFs: UiO–66 and its amine derivative, J. Radioanal. Nucl. Chem., **2016**, 307, 269–276.

[7] A. Rajaei, K. Ghani, M. Jafari, Modification of UiO–66 for removal of uranyl ion from aqueous solution by im–mobilization of tributyl phosphate, J. Chem. Sci., **2021**, 133, 14.

[8] J. Wang, Y. Sun, X. Zhao, L. Chen, S. Peng, C. Ma, G. Duan, Z. Liu, H. Wang, Y. Yuan, N. Wang, A poly(amidoxime)–modified MOF macroporous membrane for high–efficient uranium extraction from seawater, e–Polymers, **2022**, 22, 399–410.

[9] B. Zhao, L. Yuan, Y. Wang, T. Duan, W. Shi, Carboxylated UiO–66 Tailored for U(VI) and Eu(III) Trapping: From Batch Adsorption to Dynamic Column Separation, ACS Appl. Mater. Interfaces, **2021**, 13, 16300–16308.

[10] P. Yang, Q. Liu, J. Liu, H. Zhang, Z. Li, R. Li, L. Liu, J. Wang, Interfacial growth of a metal–organic framework (UiO–66) on functionalized graphene oxide (GO) as a suitable seawater adsorbent for extraction of uranium(vi), J. Mater. Chem. A, **2017**, 5, 17933–17942.

[11] S. Su, R. Che, Q. Liu, J. Liu, H. Zhang, R. Li, X. Jing, J. Wang, Zeolitic Imidazolate Framework–67: A promising candidate for recovery of uranium (VI) from seawater, Colloids Surf., A, **2018**, 547, 73–80.

[12] C. Bi, C. Zhang, W. Xu, F. Ma, L. Zhu, R. Zhu, Q. Qi, L. Liu, J. Bai, H. Dong, Highly efficient antibacterial adsorbent for recovering uranium from seawater based on molecular structure design of PCN–222 post–engineering, Desalination, **2023**, 545, 116169.

[13] Y. Song, H. Li, T. Shan, P. Yang, S. Li, Z. Liu, C. Liu, C. Shen, MOF–implanted poly (acrylamide–co–acrylic acid)/chitosan organic hydrogel for uranium extraction from seawater, Carbohydr. Polym., **2023**, 302, 120377.

[14] J. Yu, J. Wang, H. Zhang, Q. Liu, J. Liu, J. Zhu, J. Yu, R. Chen, MOF–derived Co–Ni layered double hydroxides/polyethyleneimine modified chitosan micro–nanoreactor for high–efficiency capture of uranium from seawater, Carbohydr. Polym, **2024**, 323, 121426.
